# Supplementary material for: Cerebral Cavernous Malformation 1 Determines YAP/TAZ Signaling-Dependent Metastatic Hallmarks of Prostate Cancer Cells
Source: Cancers (Basel). 2021 Mar 5;13(5):1125. doi: 10.3390/cancers13051125 (PMC7961486; doi:10.3390/cancers13051125)
Supplement: Supplementary file 1 [file cancers-13-01125-s001.zip › cancers-1068035-supplementary file 1.docx]

**Table S1.** List of qPCR primers used in this study.

| Gene Name | Forward (5’ to 3’) | Reverse (5’ to 3’) |
| --- | --- | --- |
| CCM1 | TGTTGCATGAAGTTCCCATT | GCAGGAGAAATTGGTTTGGT |
| GAPDH | TCAAGAAGGTGGTGAAGCAG | CGCTGTTGAAGTCAGAGGAG |
| 18S rRNA | CCGAAGATATGCTCATGTGG | GTGATCACACGTTCCACCTC |
| AR | CGTCTACCCTGTCTCTCTACAA | CCAGTTCATTGAGGCTAGAGAG |
| KLK2 | GCCCATTGCCTAAAGAAGAA | TGTCTTCAGGCTCAAACAGG |
| KLK3 | CAGGTCCATGACCTTCACAG | CAAGACGACTGGAACGAGAA |
| Ankrd1 | TCCGCGCCATACATAATCAGGAGT | TTAGCGCCCGAGATAAGTTGCTCA |
| Birc5 | AGCATTCGTCCGGTTGCGCT | TCGATGGCACGGCGCACTTT |
| Ctgf | TGCACCAGCATGAAGACATACCGA | TGTAATGGCAGGCACAGGTCTTGA |
| Cyr61 | TGAGTGCCGCCTTGTGAAAGAAAC | TGGGCCGGTATTTCTTCACACTCA |
| Tmprss2 | CAAGACGACTGGAACGAGAA | TCCGCTGTCATCCACTATTC |
| YAP | CTACACCCACAGCTCAGCAT | ACCAGAAGATGTCTTTGCCA |
| TAZ | ATTCATCGCCTTCCTAGGGT | GGCTGGGAGATGACCTTCAC |
| DDX5 | CAT CAA TCA TCA GCC ATT CC | CAG CTA CTT GCT GCA CCT GT |
| SLUG | ATCTGCGGCAAGGCGTTTTCCA | GAGCCCTCAGATTTGACCTGTC |
| SNAIL | TGCCCTCAAGATGCACATCCGA | GGGACAGGAGAAGGGCTTCTC |
| TWIST1 | GCCAGGTACATCGACTTCCTCT | TCCATCCTCCAGACCGAGAAGG |
| TWIST2 | GCAAGATCCAGACGCTCAAGCT | ACACGGAGAAGGCGTAGCTGAG |

**
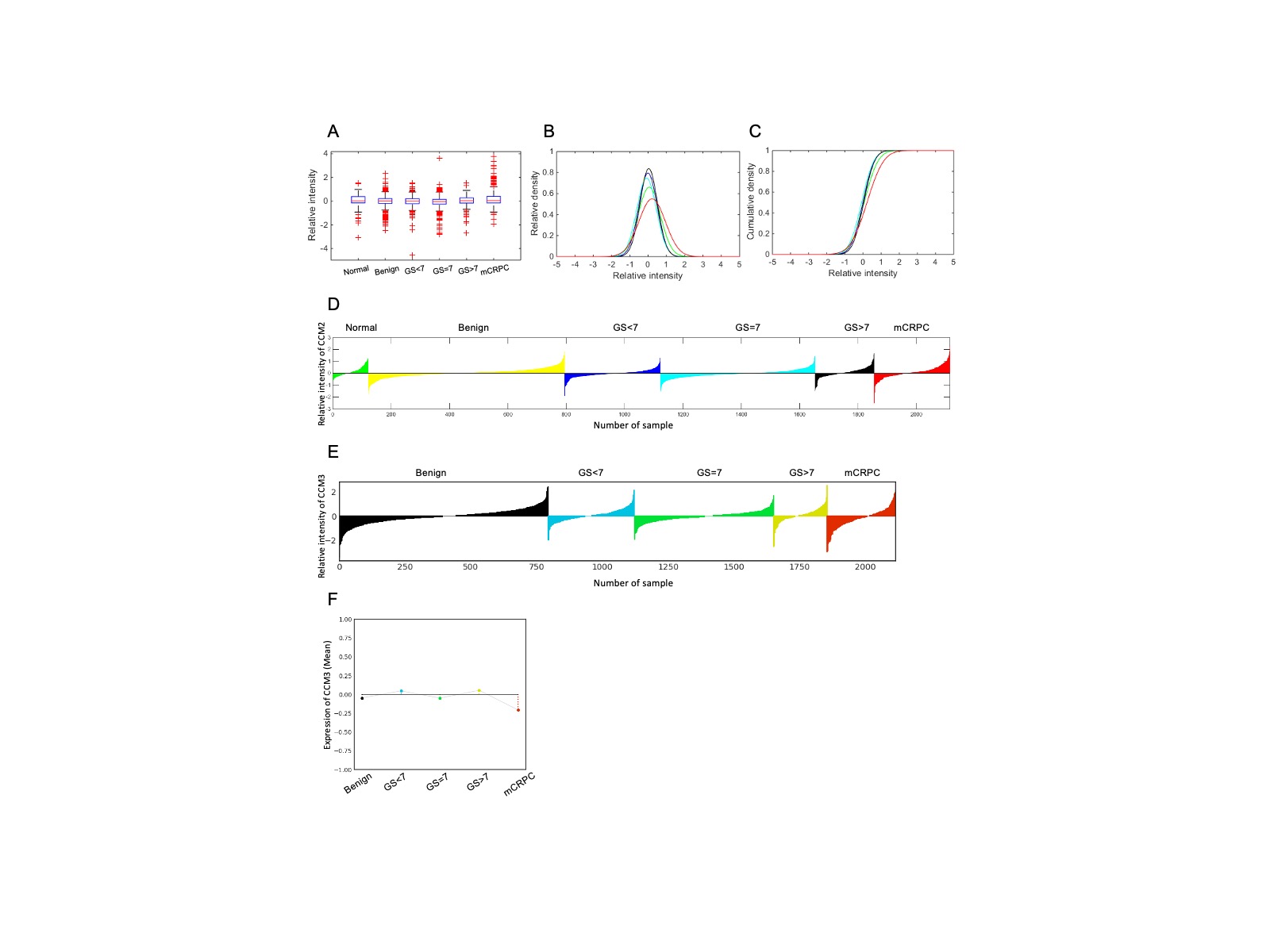
**

**Figure S1.** Expression analysis of CCM1, CCM2, and CCM3. (A) Boxplot of CCM1 gene expression in different disease states. The x-axis presents the disease state, and the y-axis presents normalized gene expression (log2 scale). (B) The distribution of samples in each disease state according to their relative CCM1 gene expression. The x-axis presents normalized gene expression, and the y-axis presents the relative density of the samples. (C) The cumulative density of the samples according to their CCM1 gene expression levels. The x-axis presents normalized gene expression, and the y-axis presents the cumulative density of the samples. Different line colors in panels B–C indicate different diseases as described for Figure 1C. (D) The waterfall plot displays the normalized CCM2 gene expression of individual samples from the prostate cancer transcriptome atlas (PCTA) cohort. (E) This waterfall plot displays the normalized CCM3 gene expression of individual samples from the PCTA cohort. (F) The stem plots with different colors show the mean expression of CCM3 in each disease state. The y-axis presents normalized gene expression (log2 scale).

**
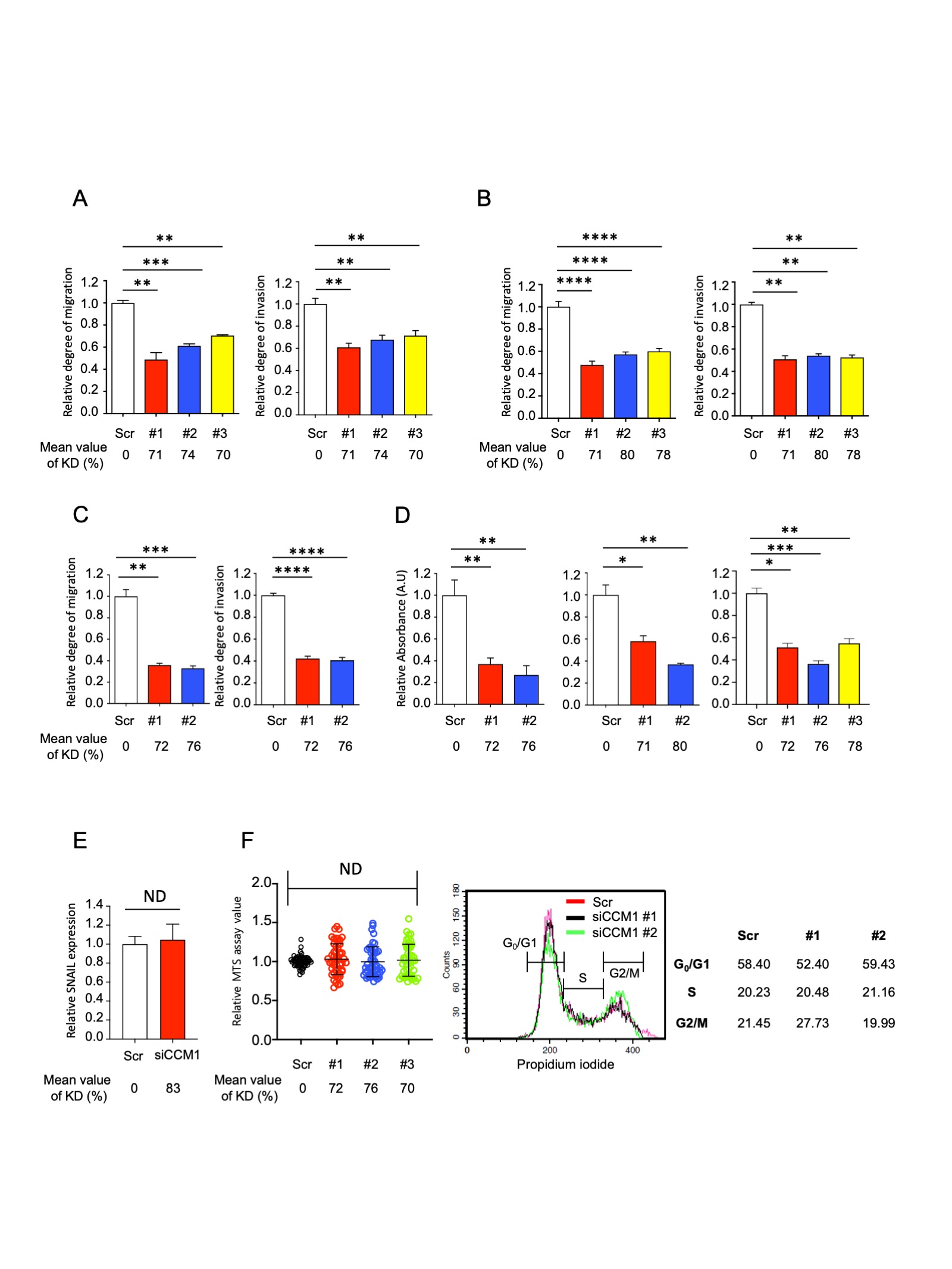
**

**Figure S2.** Suppression of CCM1 represses a metastatic hallmark in prostate cancer cells *in vitro*. (A) DU145, (B) C4-2, and (C) CWR22r shCCM1 cells were plated on Transwell membranes coated without or with Matrigel for migration (left) and invasion (right) assays, respectively, incubated for up to 72 h, and stained. (D) LNCaP (left), C4-2 (middle), and CWR22r (right) cells were grown in soft agar for 7 days, and each lysate was analyzed using a microplate reader to measure anchorage-independent survival. The data are presented as the mean ± SEM of absorbance expressed as a ratio to the expression in the Scr control. (E) SNAIL expression was analyzed via qPCR in CCM1-suppressed PC3 cells. The mean ± SEM is presented as a ratio to that in the scramble (Scr) control. t-test results (****: p <0.0001, ***: p <0.001, **: p < 0.01, *: p < 0.05). (F) Proliferation rates of LNCaP shCCM1 cells were measured with Ez-Cytox assays for 3 hrs (left). Scatter plots present the ratio of endpoint values (n = 3–5/set) from five independent experiments relative to the scramble (Scr) control mean values in each corresponding experiment. Two-way ANOVA and Turkey’s post-hoc tests were performed. ND: Not significantly different (p>0.05). Flow cytometry analysis with CCM1 suppressed LNCaP cells with siRNA did not show a remarkable difference in cell cycle progression (middle and right). Numbers indicate percentages of cells in G_0_/G1, S and G2/M phases (right).


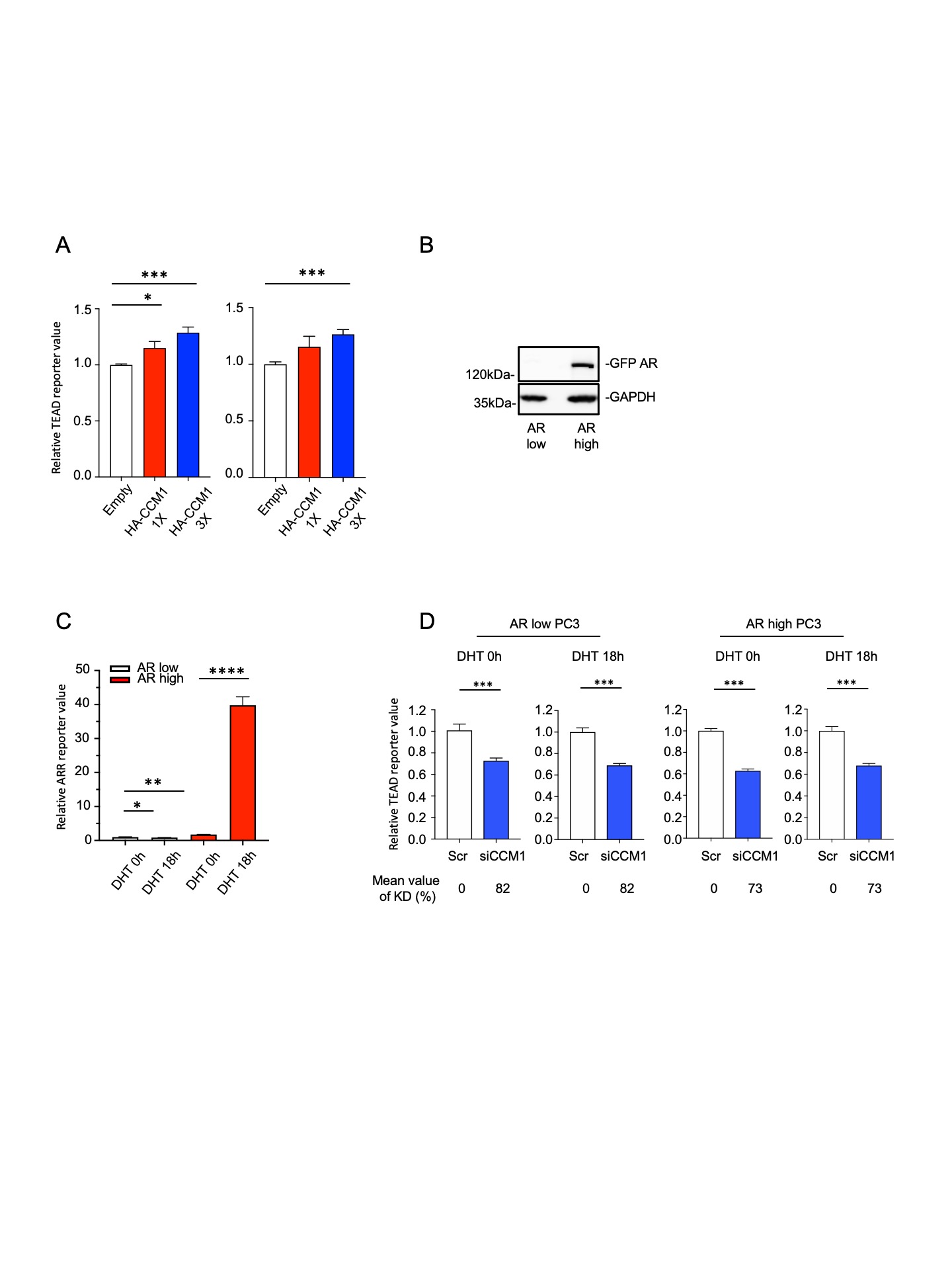


**Figure S3.** CCM1-mediated regulation of YAP/TAZ signaling. (A) PC3 (left) and LNCaP (right) cells were co-transfected with increasing amounts of CCM1 overexpression plasmids and the same amounts of TEAD reporter plasmids and grown in normal medium. TEAD reporter activity was measured using luciferase assays. (B) Androgen receptor (AR) overexpression plasmid was stably transfected in WT PC3 cells, and individual PC3 clones were sorted into AR high and AR low groups according to the expression level of ectopic AR. Expression levels of GFP-AR from representative AR high or low PC3 cells are shown. Uncropped Blots of Figure S3B can be found in Supplementary File 2 (C) PC3 AR high (red box) or low (white box) cells were grown in medium supplemented with 10% charcoal-stripped FBS and stimulated with 1 µM DHT for the indicated time points, and ARR reporter activities were measured using luciferase assays. Ratio of reporter activities is shown relative to that in the DHT untreated AR low cells. (D) CCM1 was RNAi-silenced in AR low (left two graphs) or AR high (right two graphs) PC3 cells grown in 10% charcoal-stripped FBS-supplemented medium with or without DHT stimulation as indicated, and TEAD reporter activity was analyzed. t-test results (****: p <0.0001, ***: p <0.001, **: p < 0.01, *: p < 0.05).

**
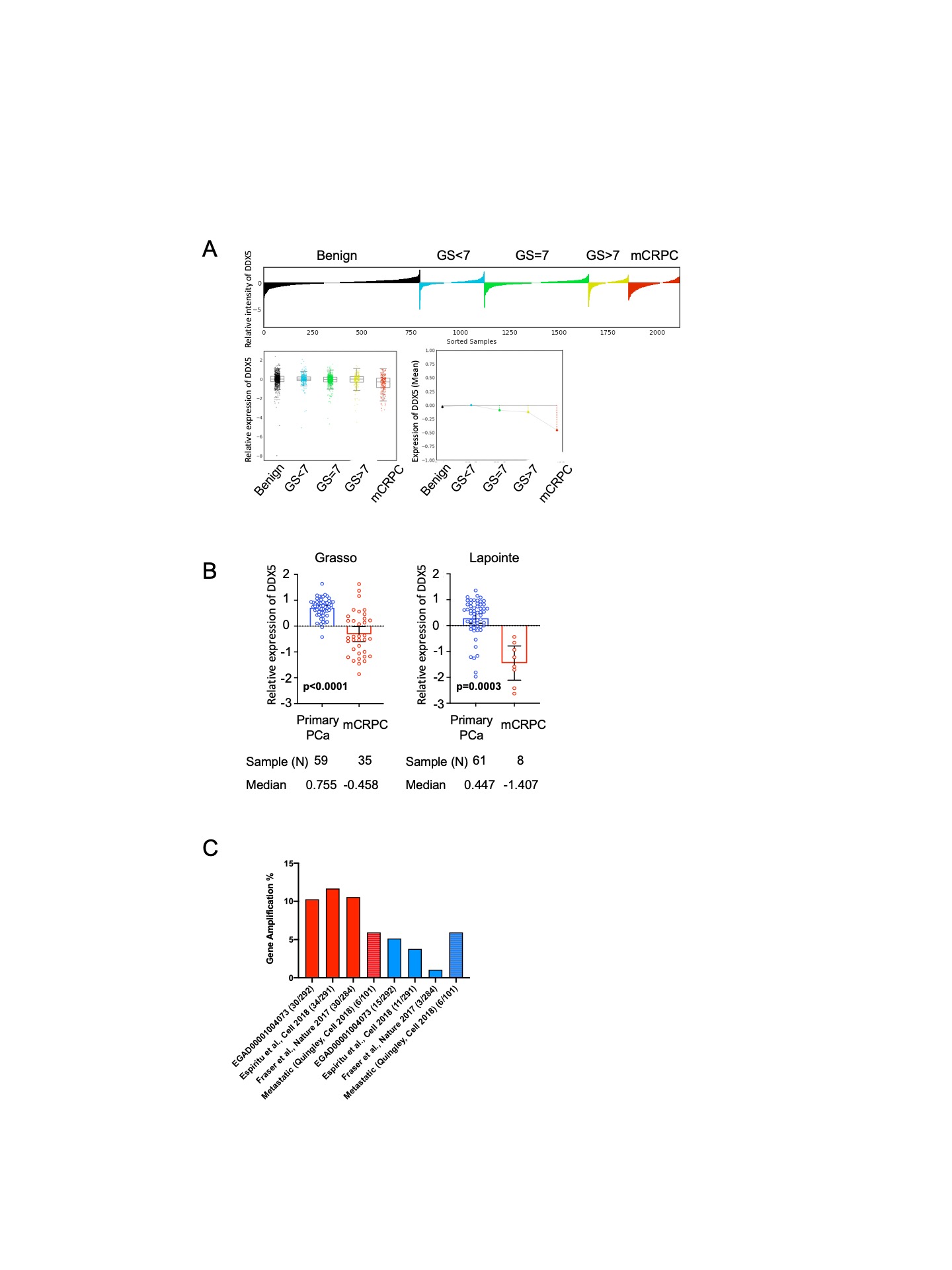
**

**Figure S4.** Expression analysis of DDX5. (A) The waterfall plot displays normalized DDX5 gene expression (log2 scale) for individual samples from the prostate cancer transcriptome atlas cohort (top). Benign: benign prostatic hyperplasia. The Wilcoxon rank sum test results between subsets (mCRPC vs. primary PCa): fold change = −0.383, P < 0.001. (bottom left) Boxplot of DDX5 gene expression in different disease states. The x-axis presents the disease state, and the y-axis presents normalized gene expression. (bottom right) The stem plots with different colors show the mean expression levels of DDX5 in each disease state. The y-axis values represent normalized expression of DDX5. Different colors in panel B indicate different diseases as described. (B) Scatter plot analysis of the relative expression of DDX5 between primary PCa and metastatic CRPC samples from two transcriptome studies (Grasso [42] and Lapointe [43] studies). Y-axis values, which were retrieved from the Oncomine database, are shown as the log2 median-centered ratio. Mean with 95% confidence intervals. t-test was used for statistical significance. (C) Shown are comparisons of the frequencies of the gene amplification of CCM1 (red bars) and DDX5 (blue bars) from four independent cohort studies. In each group of four bars, the first three bars represent data from primary prostate cancer studies, and the fourth bar represents data from a metastatic castration-resistant prostate cancer (mCRPC) study. The y-axis presents percentiles (100 × [number of samples with gene amplification/number of total samples]).


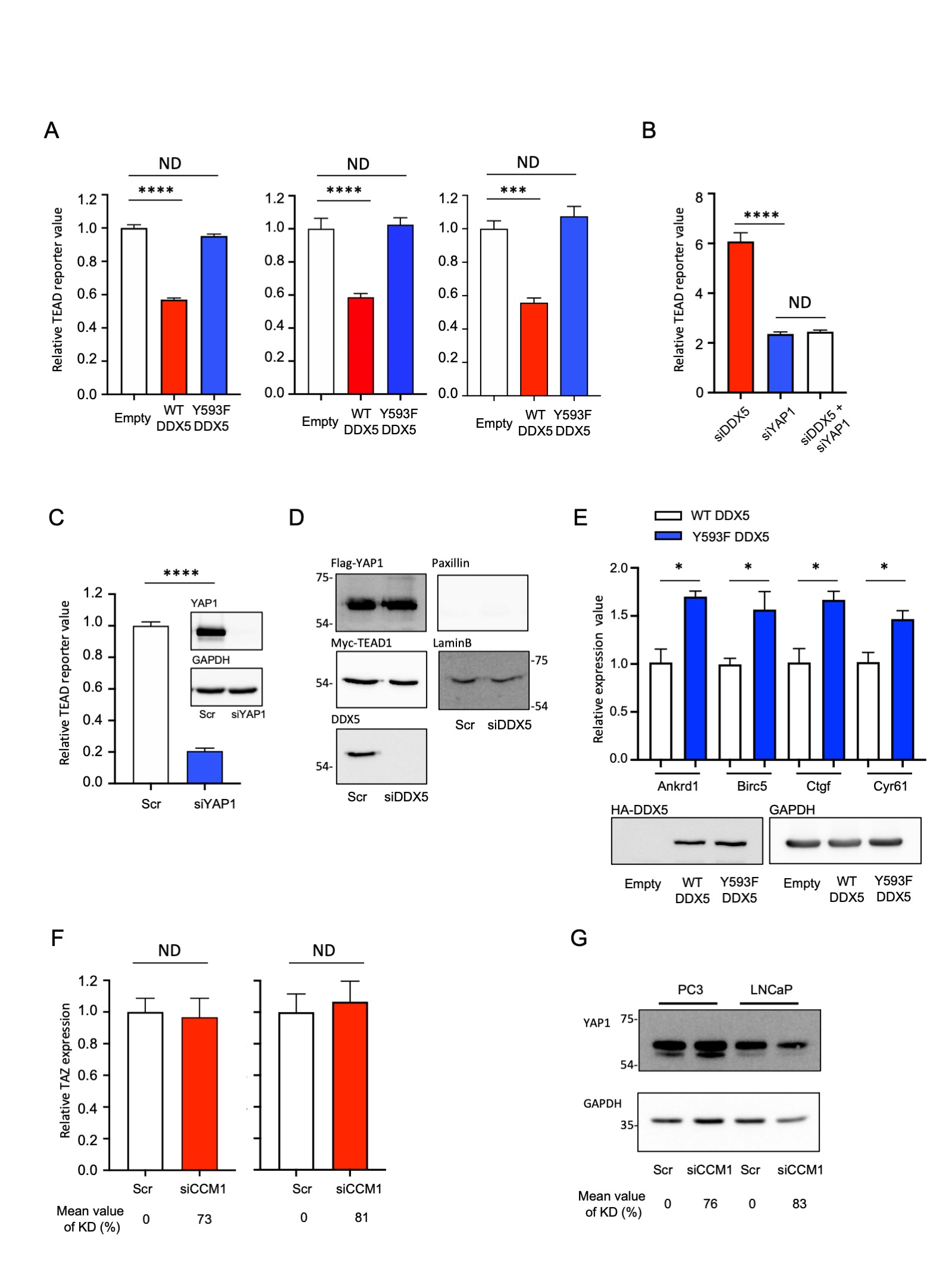


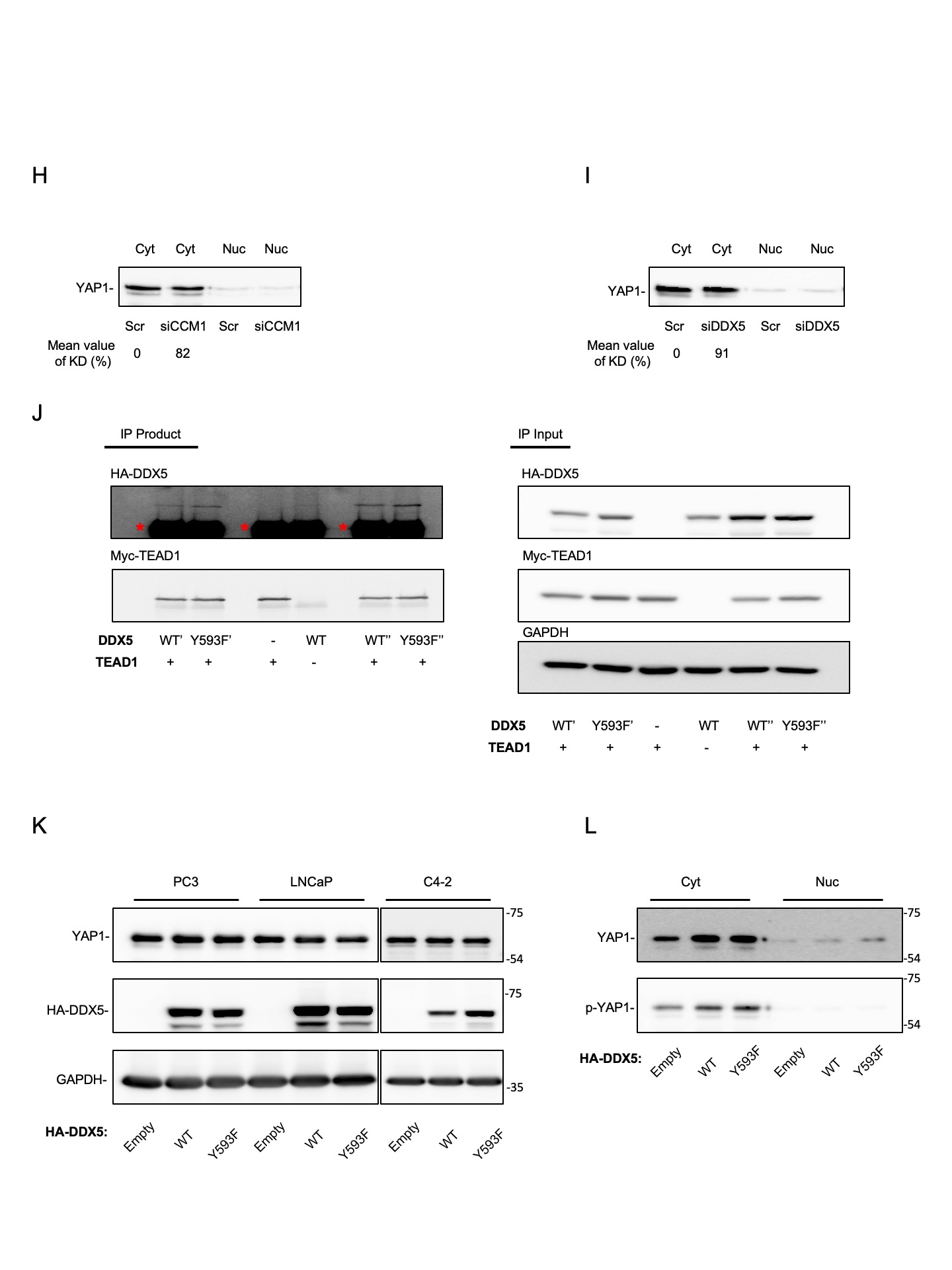


**Figure S5.** DDX5 regulates YAP/TAZ signaling. (A) WT or Y593F DDX5 was overexpressed in LNCaP (left), C4-2B (middle), and CWR22r (right) cells, and TEAD reporter activity was measured. The mean ± SEM is presented as a ratio to that in the empty plasmid transfection control. (B) RNAi-mediated suppression of DDX5 or YAP1 alone or co-suppression of DDX5 and YAP1 was performed, and TEAD reporter activity was analyzed in C4-2 cells grown in medium supplemented with charcoal-stripped FBS and stimulated with DHT for 18 h. The mean ± SEM is presented as a ratio to that in the scramble (Scr) control (not shown). Uncropped Blots of Figure S5C can be found in Supplementary File 2. (C) YAP1 was suppressed via RNAi in LNCaP cells, and TEAD reporter activity was analyzed. Suppression of YAP1 was confirmed via immunoblotting (Inset).

(D) PC3 cells co-transfected with overexpression plasmids for Flag-YAP, Myc-TEAD1, and siDDX5, were harvested, and nuclear lysates were generated. Shown are immunoprecipitation input samples from the nuclear lysates used in Figure 5G. Paxillin, a cytosolic marker, is not shown in these nuclear lysates. Uncropped Blots of Figure S5D can be found in Supplementary File 2. (E) WT DDX5 or Y593 DDX5 was overexpressed in LNCaP cells and mRNA levels of representative YAP/TAP target genes were characterized. Shown are levels of overexpressed WT DDX5 or Y593F DDX5 in the LNCaP cells used (bottom panel). Uncropped Blots of Figure S5E can be found in Supplementary File 2. (F) CCM1 was suppressed via RNAi in PC3 (left) and LNCaP (right), and mRNA level of TAZ was analyzed. (G) YAP1 expression was analyzed in the total lysates of CCM1-suppressed PC3 and LNCaP cells. Uncropped Blots of Figure S5G can be found in Supplementary File 2. (H, I) Subcellular localization of YAP1 was analyzed in CCM1-suppressed (H) and DDX5-suppressed PC3 cells (I). Uncropped Blots of Figure S5H can be found in Supplementary File 2. Uncropped Blots of Figure S5I can be found in Supplementary File 2. (J) HA-tagged WT or Y593F DDX5 and myc-tagged TEAD1 were co-expressed in C4-2 cells. The total lysates were immunoprecipitated with anti-myc antibodies, and immunoblotted with anti-myc or anti-HA antibodies to detect immunoprecipitated myc-TEAD1 and co-immunoprecipitated HA-DDX5, respectively (left panel). Shown are our co-immunoprecipitation assays replicated (′, ″). Levels of overexpressed HA-tagged DDX5 and myc-tagged TEAD1 in the immunoprecipitation input samples (right panel). Red asterisks: heavy chain bands. Uncropped Blots of Figure S5J can be found in Supplementary File 2. (K) HA-tagged WT or Y593F DDX5 were overexpressed in PC3, LNCaP, and C4-2 cells, and YAP1 levels in each total lysate were analyzed via immunoblotting. Uncropped Blots of Figure S5K can be found in Supplementary File 2. (L) Cytosolic and nuclear fractions of C4-2 cells expressing ectopic WT or Y593F DDX5 were separated, and the endogenous levels of total YAP1 and S127-phosphorylated YAP1 (p-YAP1) in cytosolic and nuclear fractions were analyzed. Uncropped Blots of Figure S5L can be found in Supplementary File 2. Cyt: cytosolic fraction, Nuc: nuclear fraction. t-test results (****: p <0.0001, ***: p <0.001, **: p < 0.01, *: p < 0.05, ND: Not significantly different (p>0.05)).

**
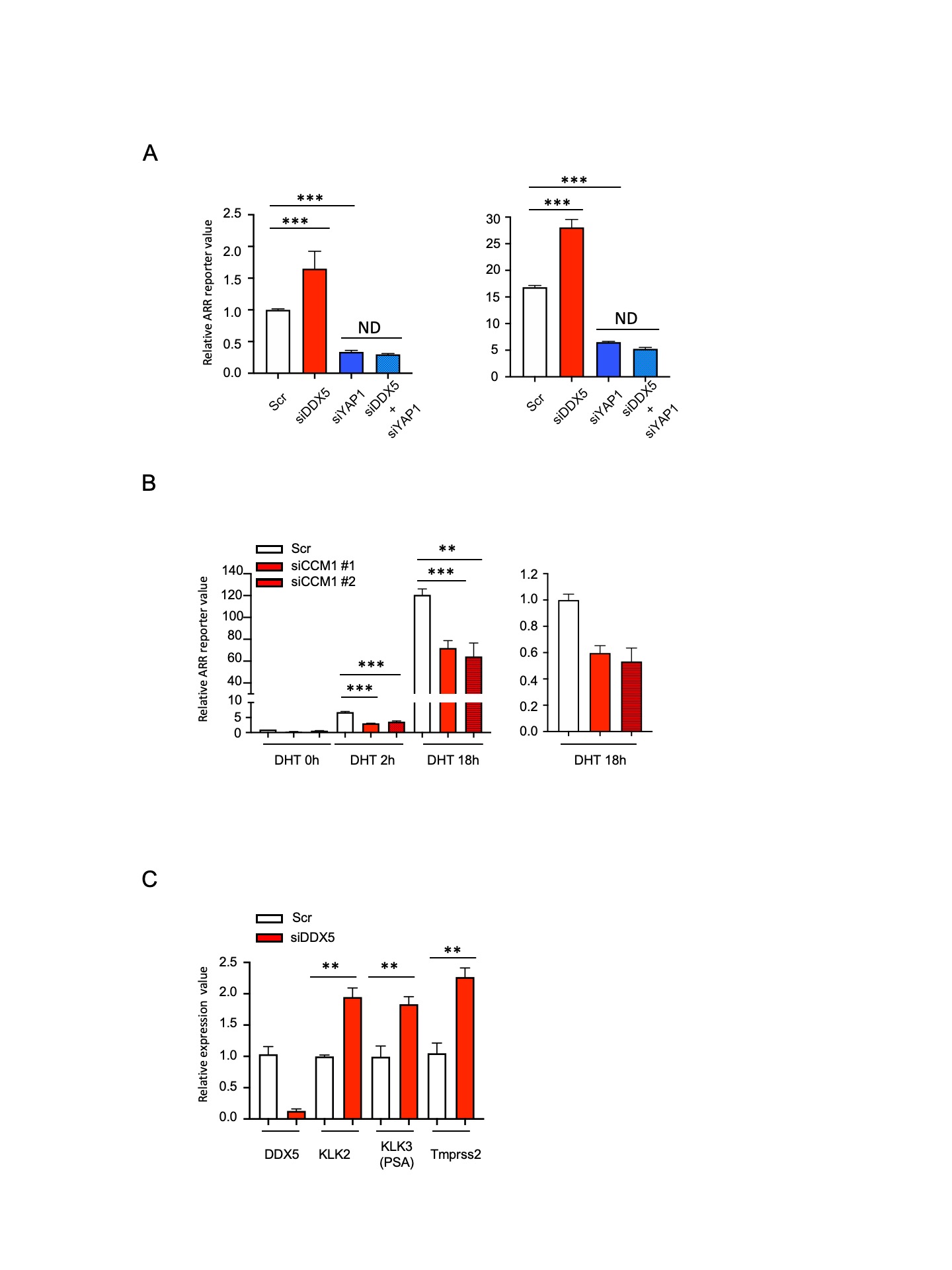
**

**Figure S6.** Regulatory mechanisms of DDX5 and CCM1 in AR signaling. (A) RNAi-mediated silencing of DDX5 or YAP1 or co-silencing of DDX5 and YAP1 and transfection of ARR reporter plasmids were performed. LNCaP cells were grown in charcoal-stripped FBS-supplemented medium and stimulated without (left) or with 1 µM DHT (right), and ARR reporter activity was analyzed. (B) LNCaP cells were co-transfected with two different CCM1 siRNAs (#1, #2) and ARR reporter plasmids, grown in charcoal-stripped FBS-supplemented medium, and stimulated with 1 µM DHT for the indicated times. Ratio of reporter activities in CCM1 silenced cells relative to that in the scramble control after 18 h of DHT treatment (right). The mean ± SEM is presented as a ratio to that in the scramble (Scr) control without DHT stimulation. (C) DDX5 was suppressed with RNAi in LNCaP cells grown in normal media and mRNA level of DDX5 and representative AR target genes were measured. t-test results (****: p <0.0001, ***: p <0.001, **: p < 0.01, *: p < 0.05, ND: Not significantly different (p>0.05)).
