# Supplementary material for: Cerebral Cavernous Malformation 1 Determines YAP/TAZ Signaling-Dependent Metastatic Hallmarks of Prostate Cancer Cells
Source: Cancers (Basel). 2021 Mar 5;13(5):1125. doi: 10.3390/cancers13051125 (PMC7961486; doi:10.3390/cancers13051125)

Cerebral cavernous malformation 1 determines YAP/TAZ signaling-dependent metastatic hallmarks of prostate cancer cells

1. Shown are representative images of our functional assays in figure 2A, 2B and 4F.

Figure 2A

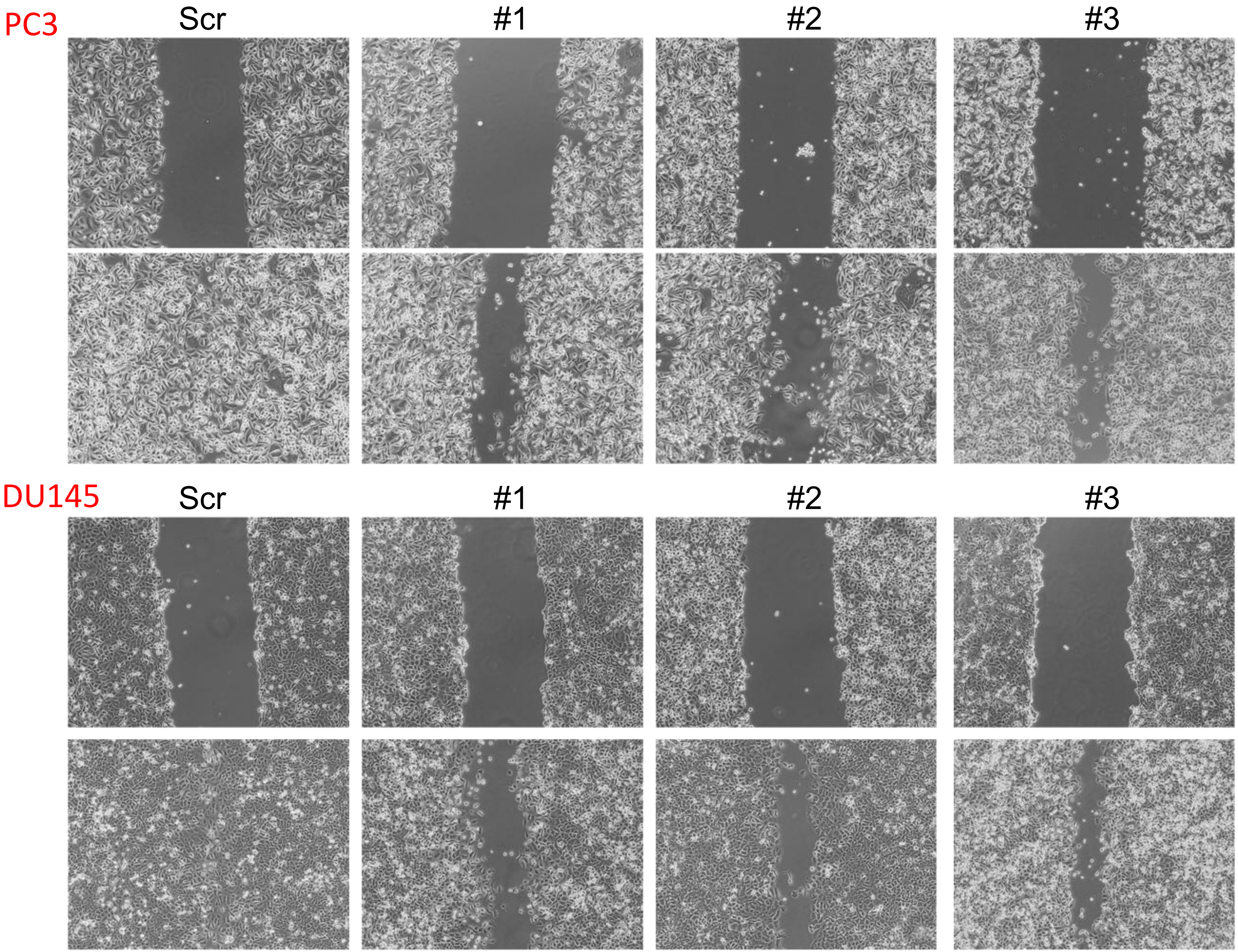

Figure 2B

PC3

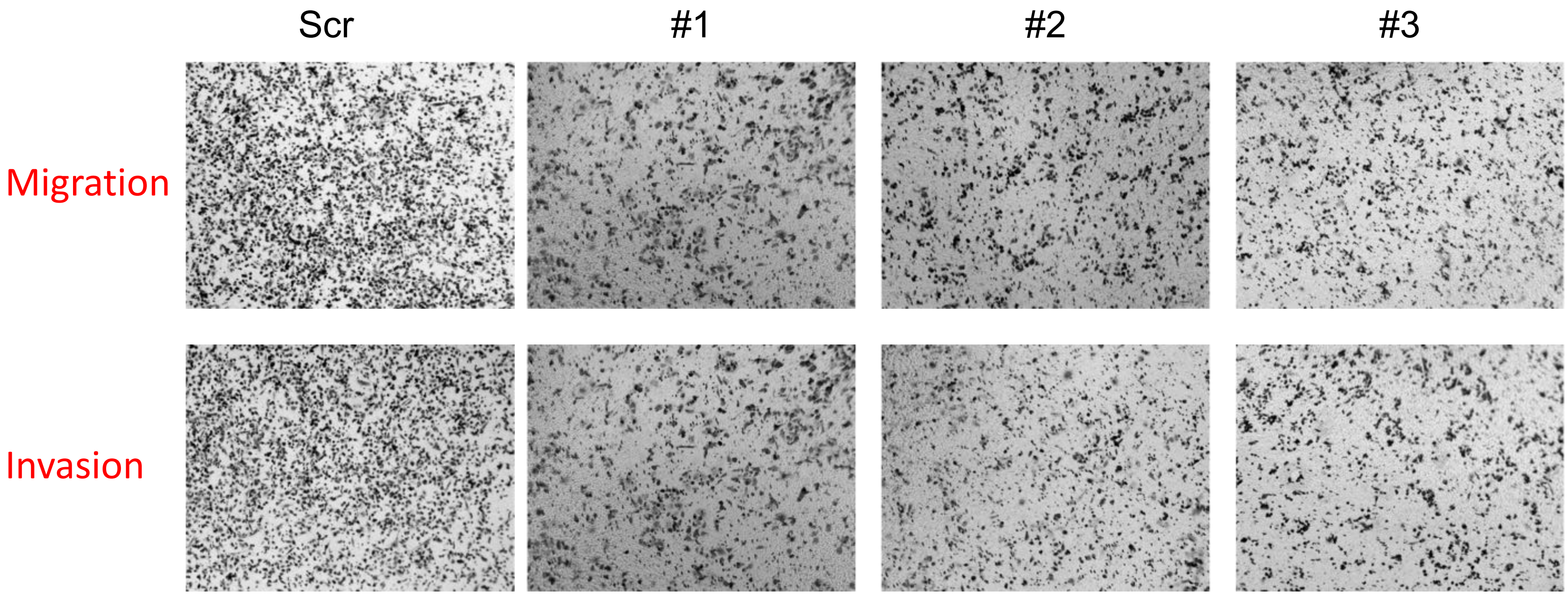

Figure 4F

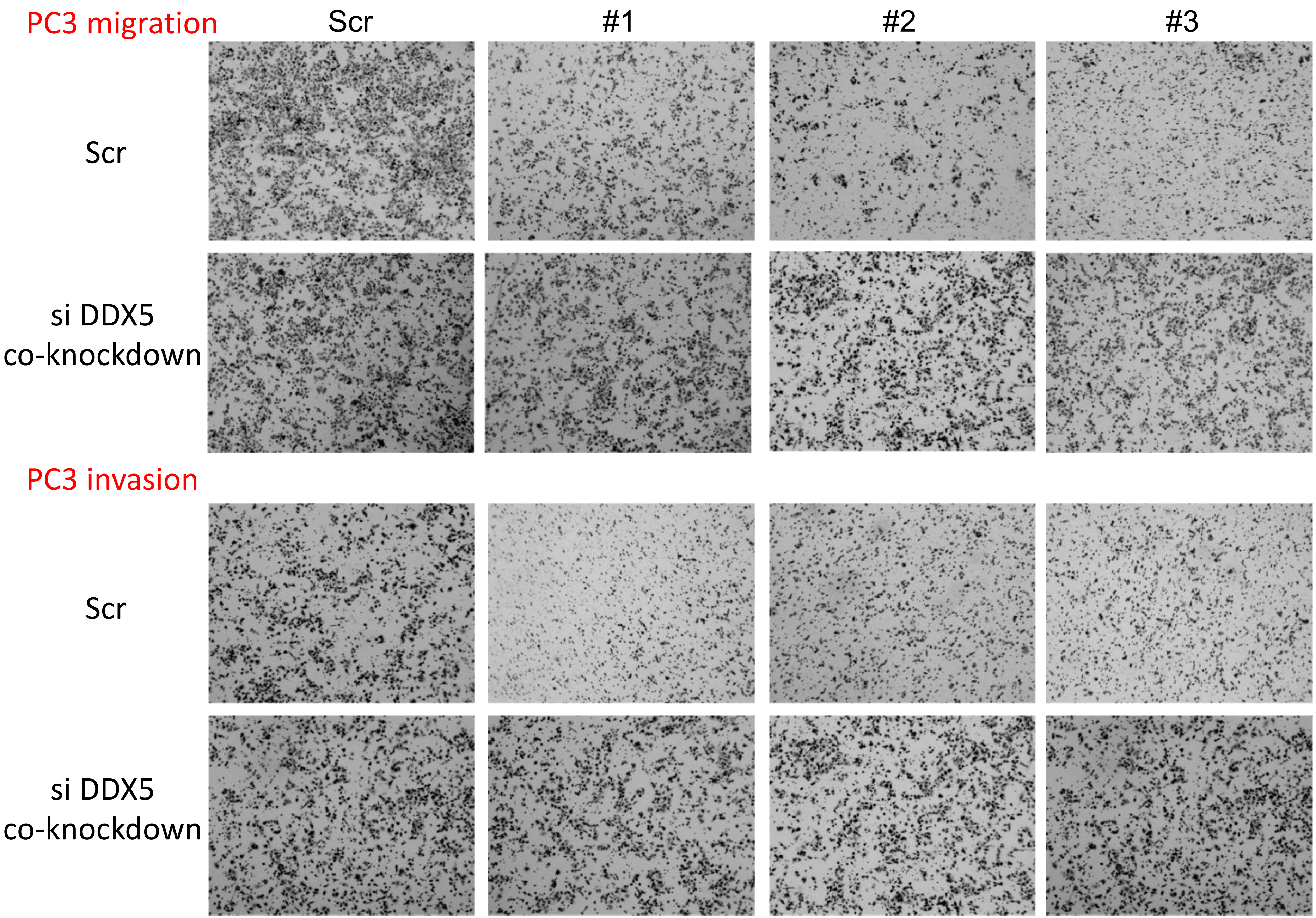
PC3 migration
Scr
#1
#2
#3
Scr
si DDX5
co-knockdown
PC3 invasion
Scr
si DDX5
co-knockdown

2. Full gel pictures.

In our study, many membranes were cut into slices or stripped to probe multiple antibodies, when necessary for comparisons including normalization.

When the boundary of each membrane slice is not clear due to short exposure from high band signals, we show extra-space enough to include the slices.

Figure 2C

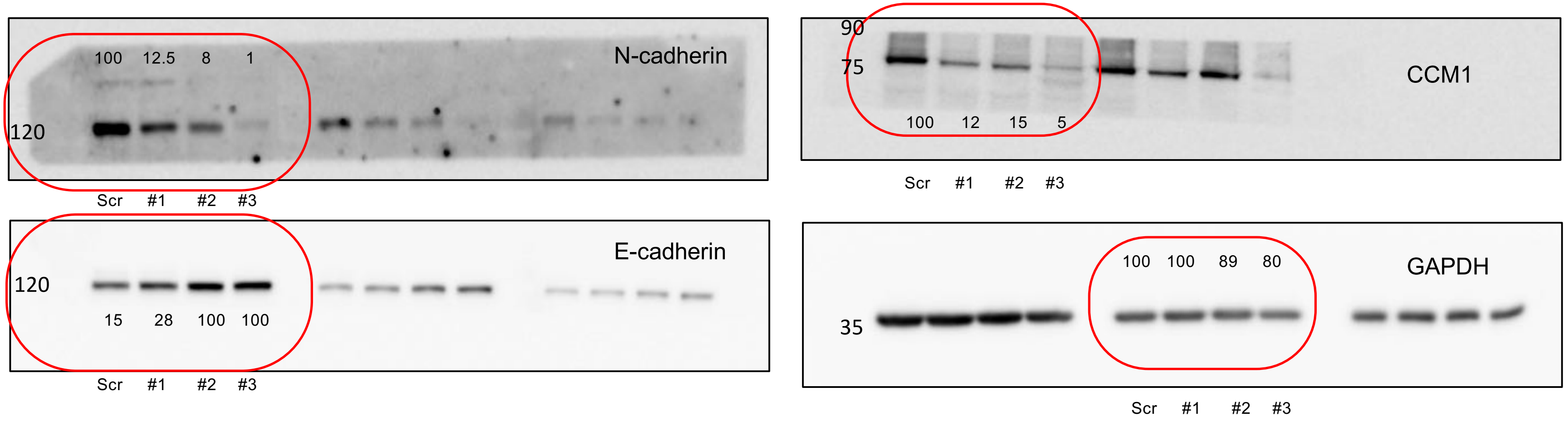

Figure 3E

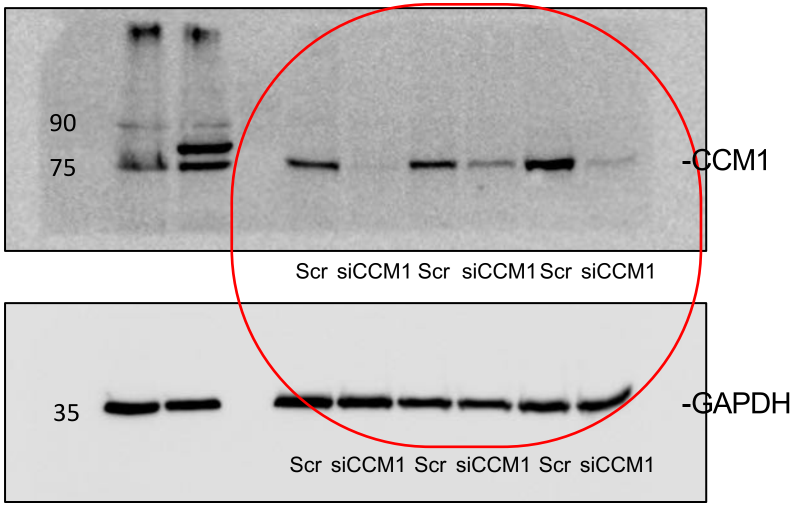

Figure 4A

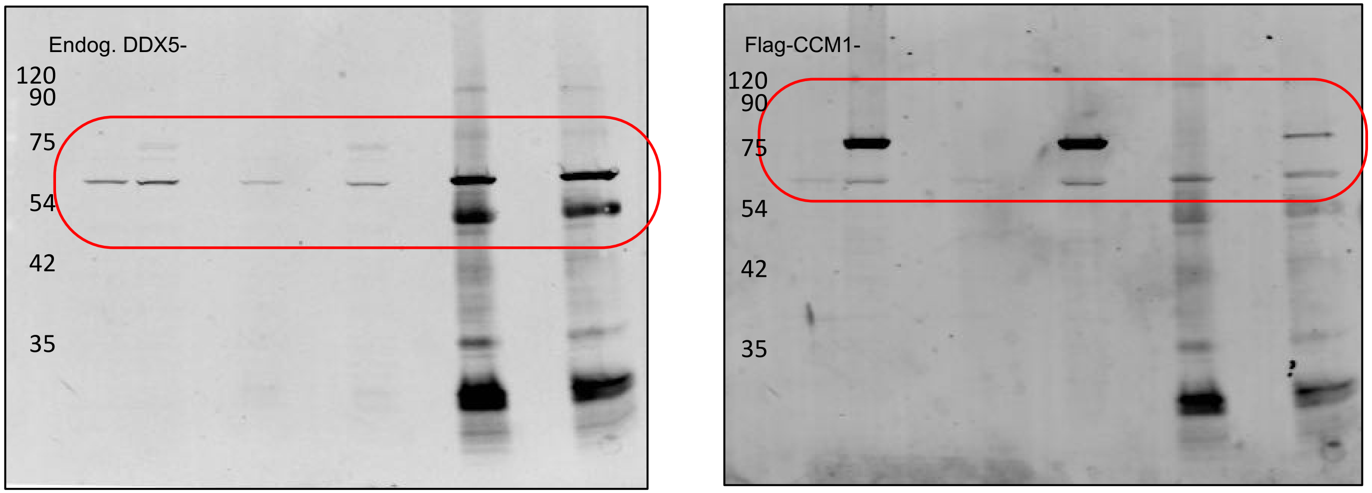

Figure 4B

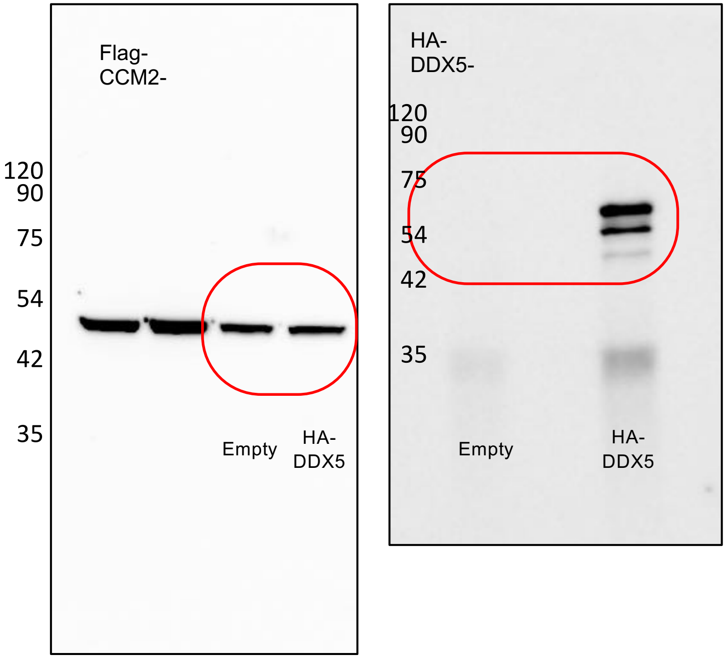

Figure 4C

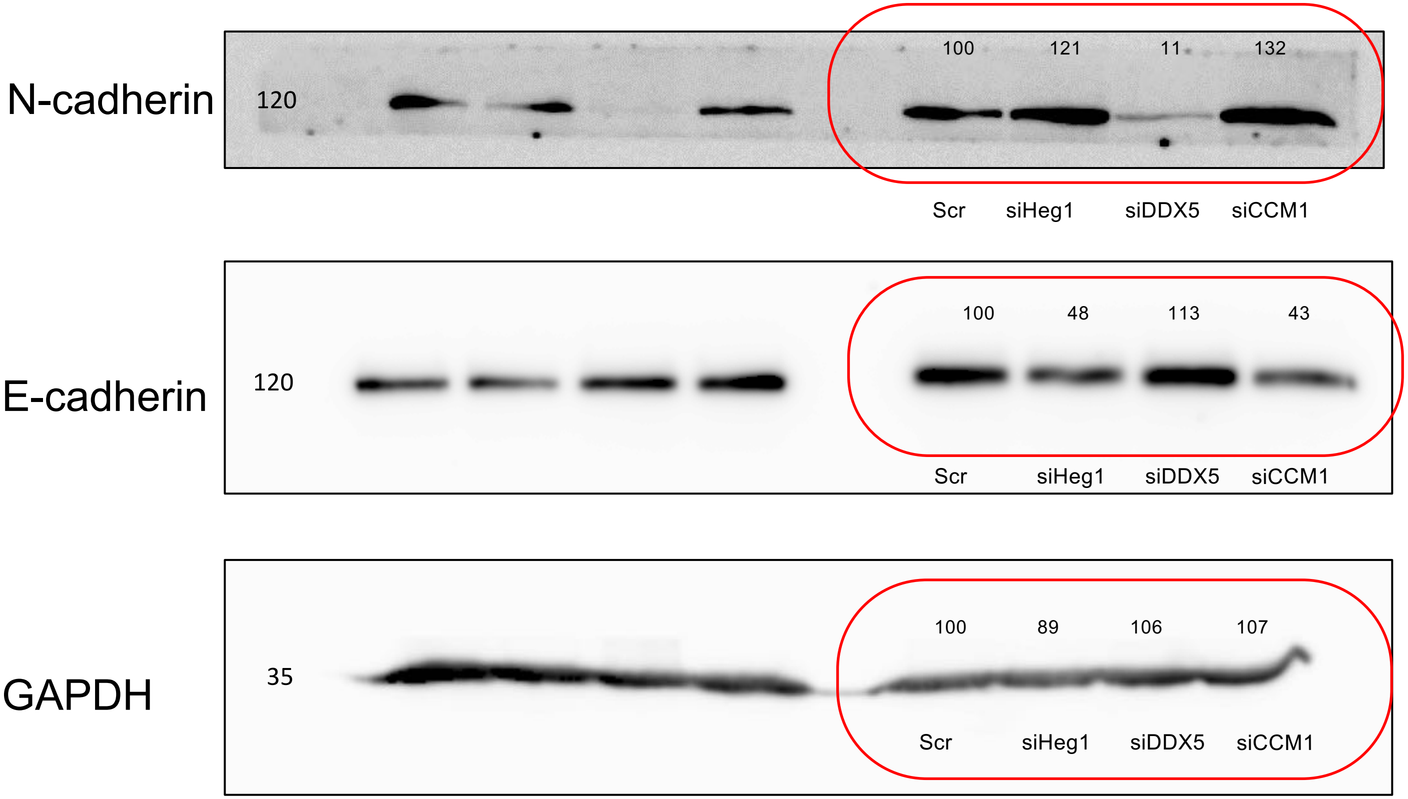
N-cadherin
120
100
121
11
132
Scr
siHeg1
siDDX5
siCCM1
E-cadherin
120
100
48
113
43
Scr
siHeg1
siDDX5
siCCM1
GAPDH
35
100
89
106
107
Scr
siHeg1
siDDX5
siCCM1

Figure 4E

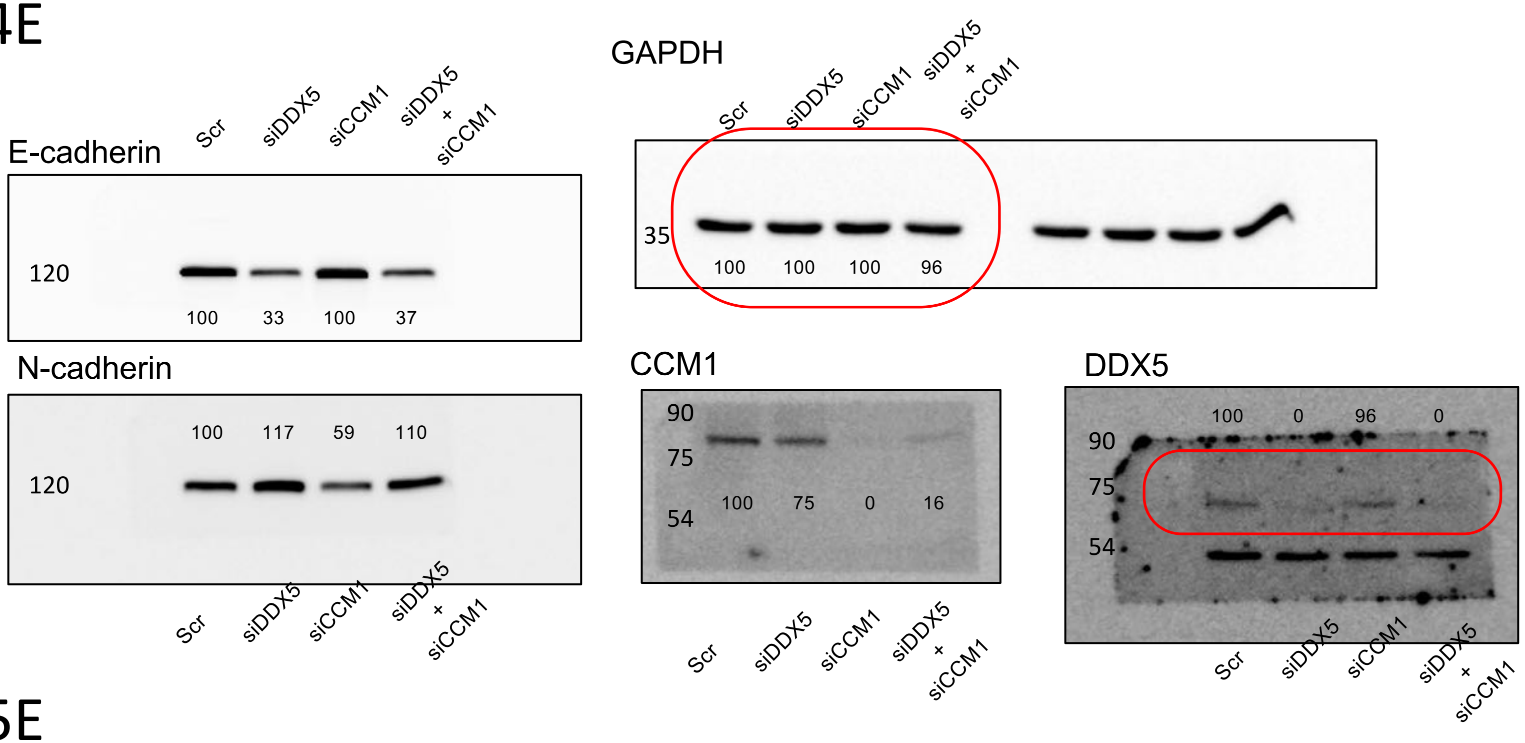

Figure 5E

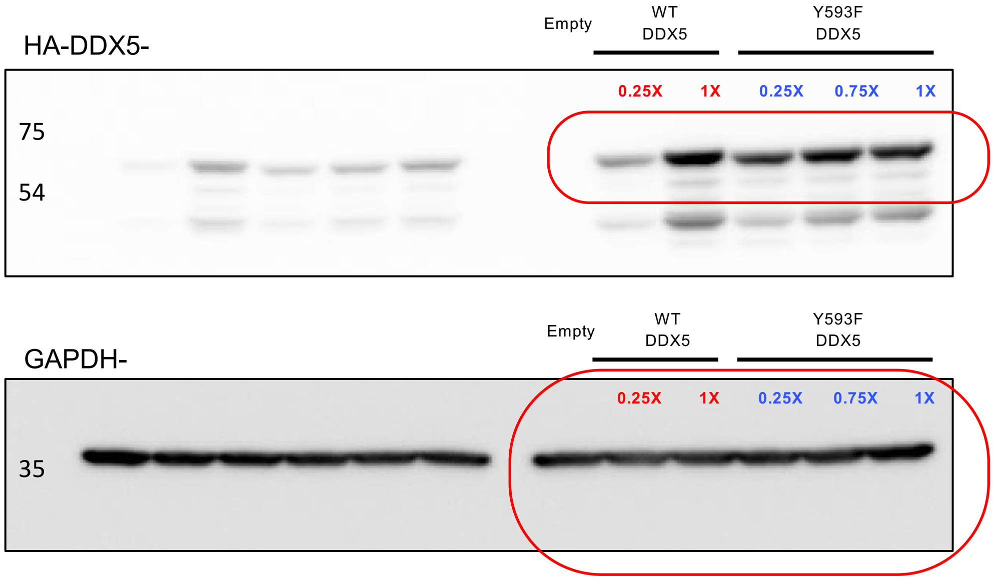

Figure 5G

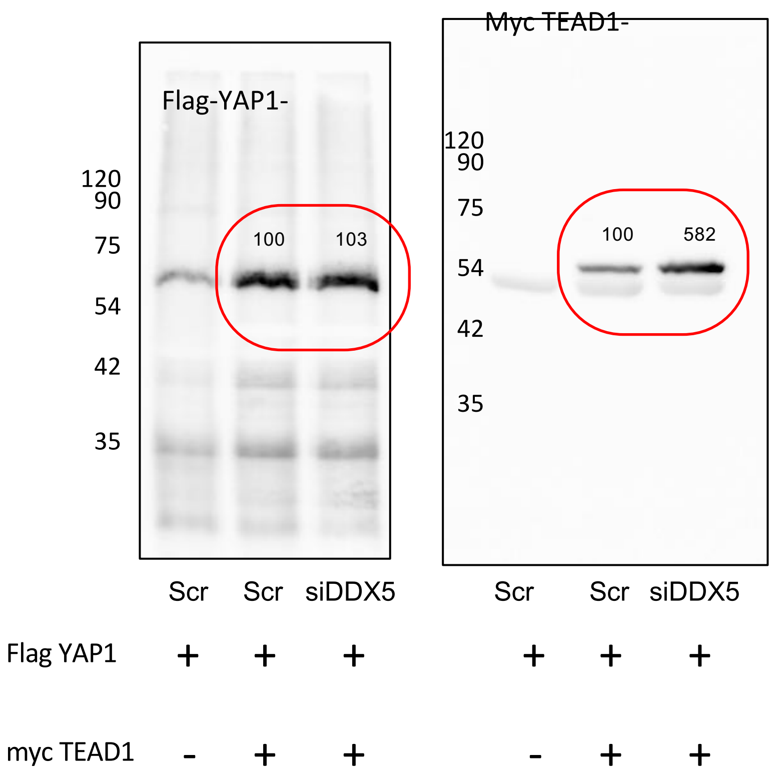

Figure 6A

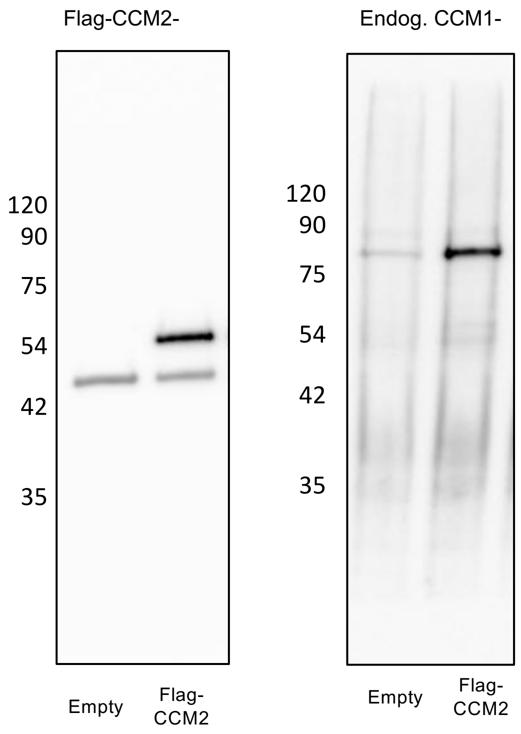

Figure 6B

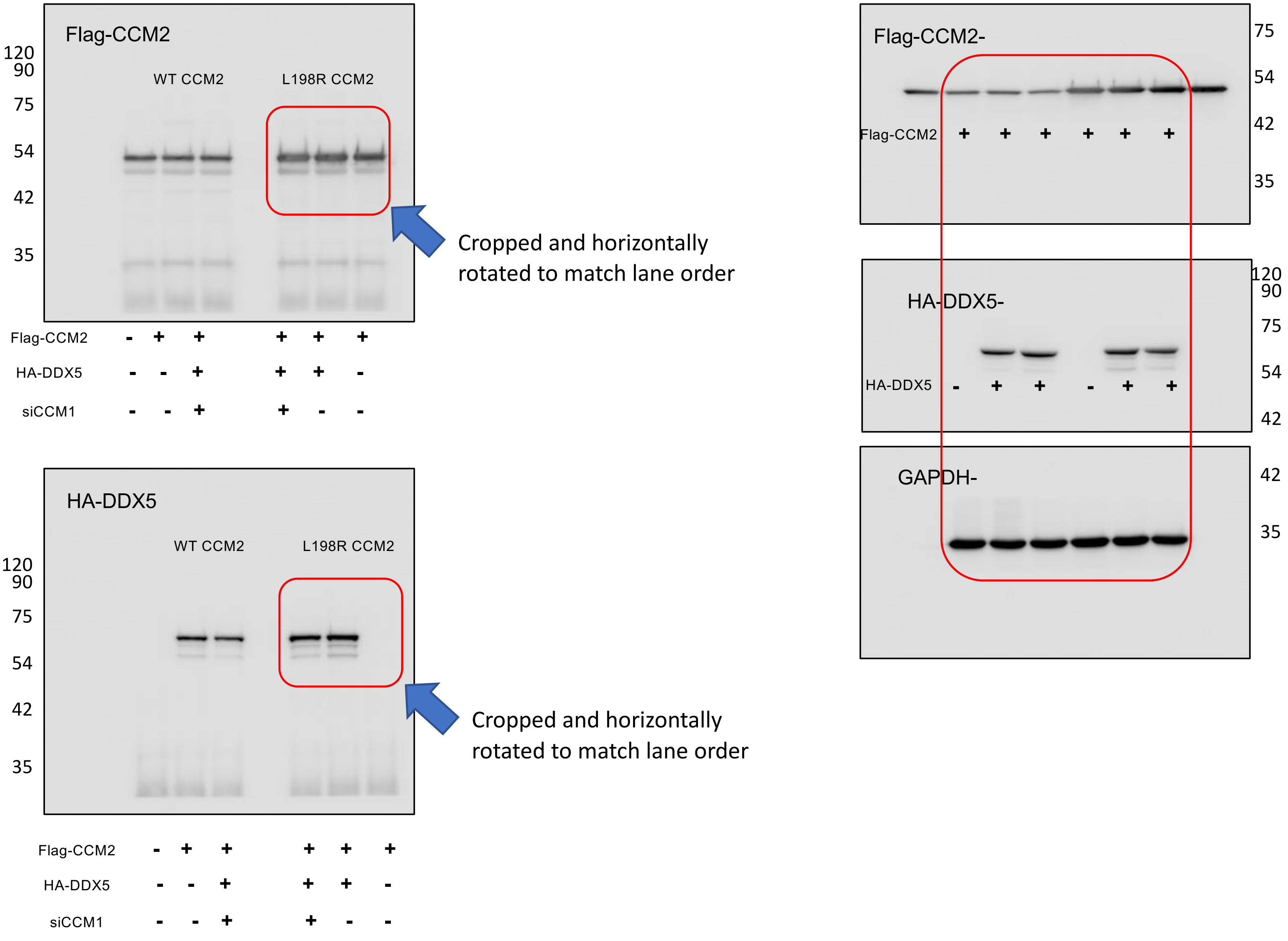
Flag-CCM2
120
90
WT CCM2
L198R CCM2
75
54
42
35
Flag-CCM2 - + + + + +
HA-DDX5 - - + + + -
siCCM1 - - + + - -
Cropped and horizontally rotated to match lane order
HA-DDX5
WT CCM2
L198R CCM2
120
90
75
54
42
35
Cropped and horizontally rotated to match lane order
Flag-CCM2 - + + + + +
HA-DDX5 - - + + + -
siCCM1 - - + + - -
Flag-CCM2-
75
54
42
35
Flag-CCM2 + + + + + +
HA-DDX5-
120
90
75
54
42
HA-DDX5 - + + - + +
GAPDH-
42
35

Figure 6C

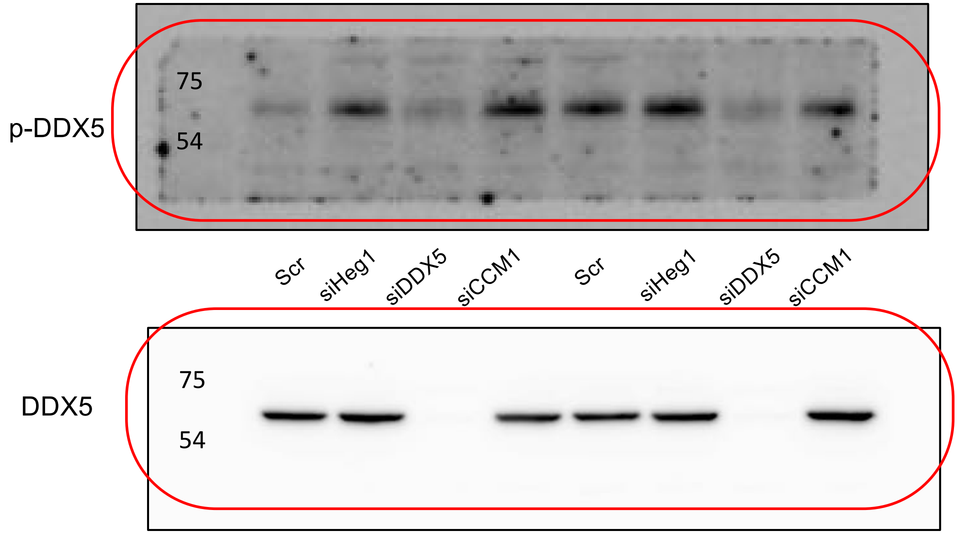

Figure 6D

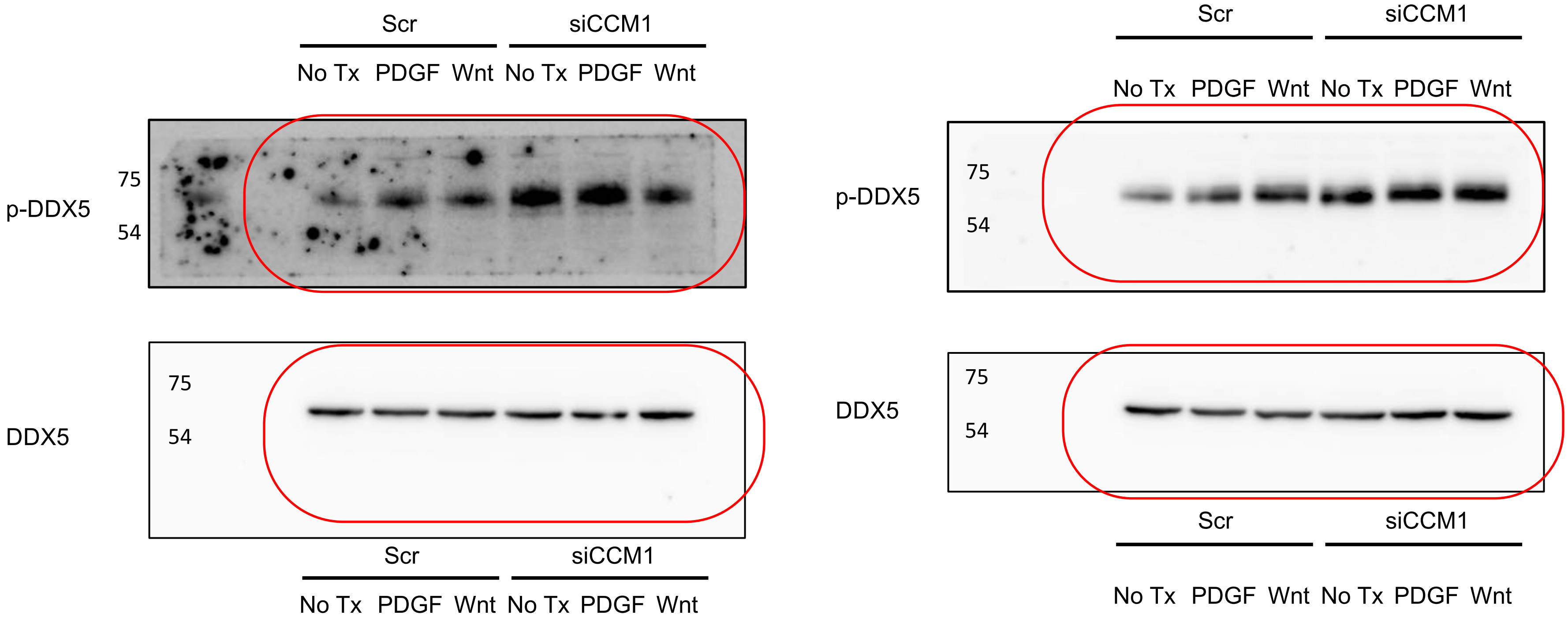

Figure S3B

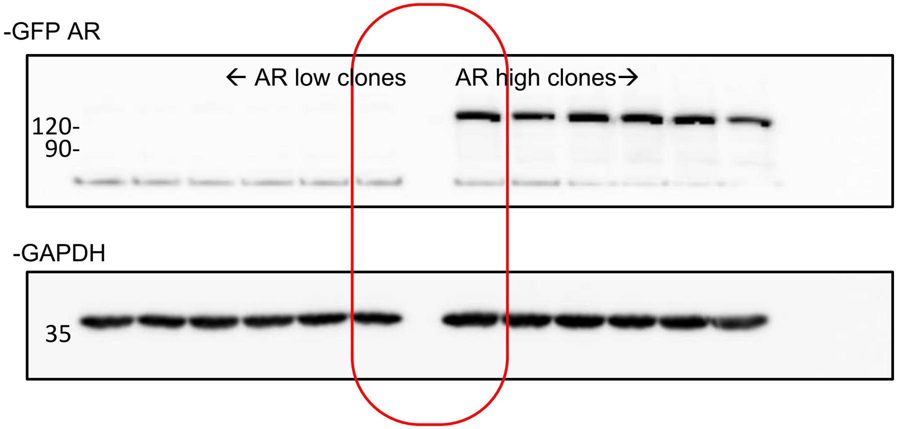

Figure S5C

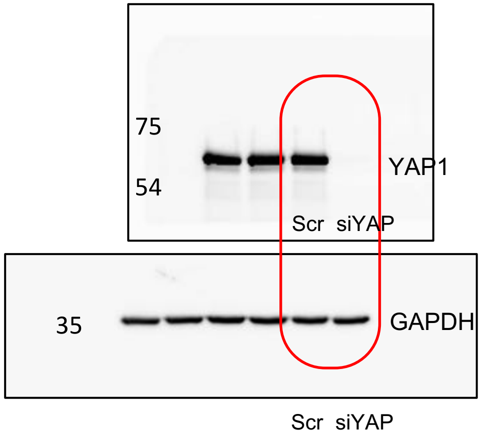

Figure S5D

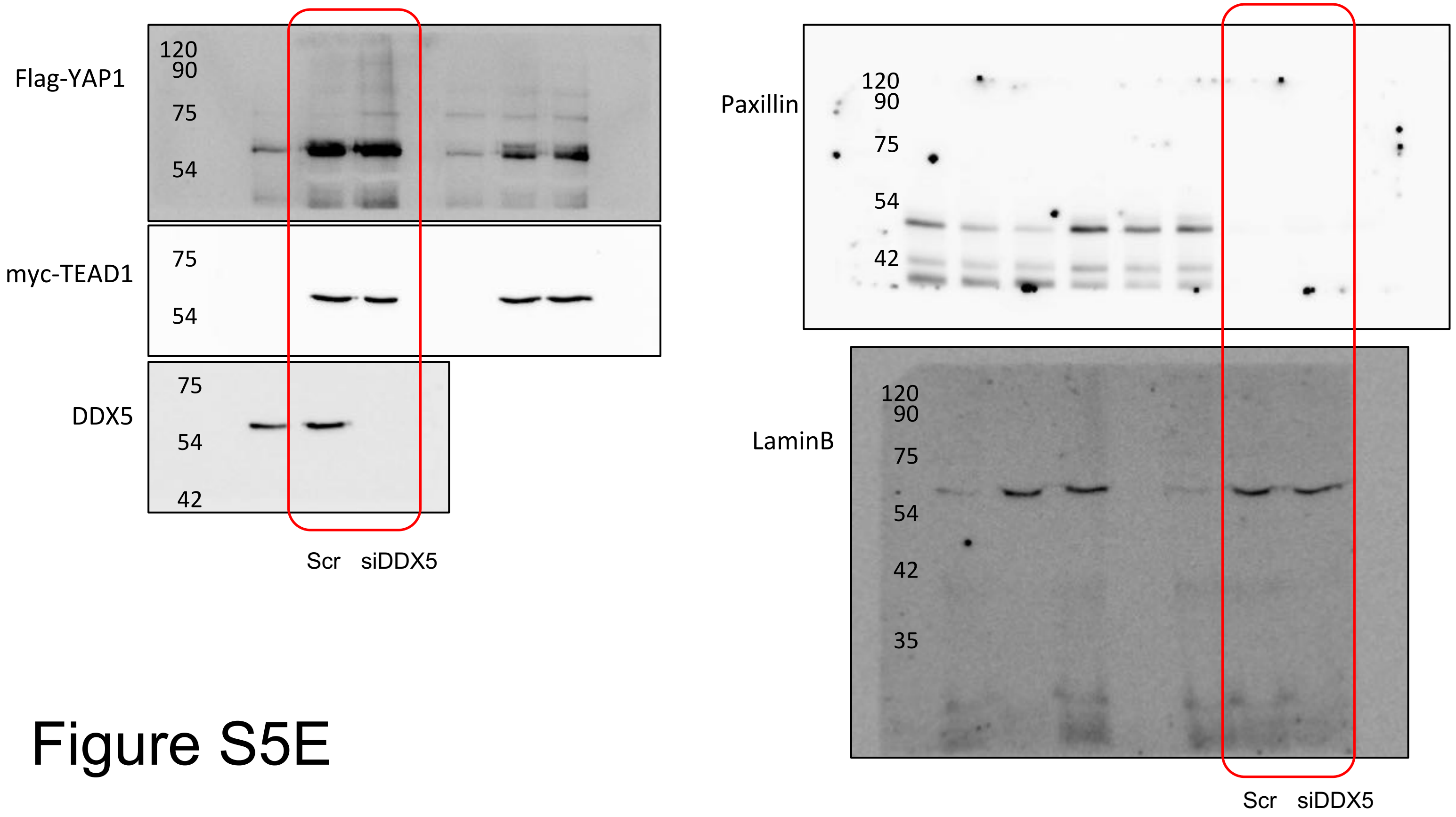

Figure S5E

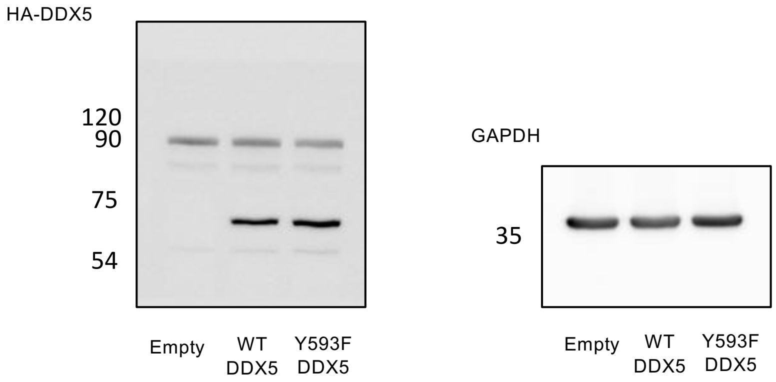

Figure S5G

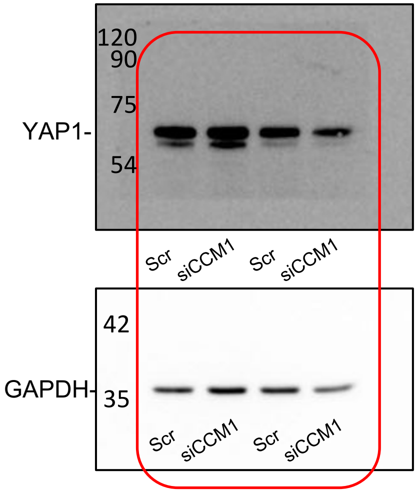

Figure S5J

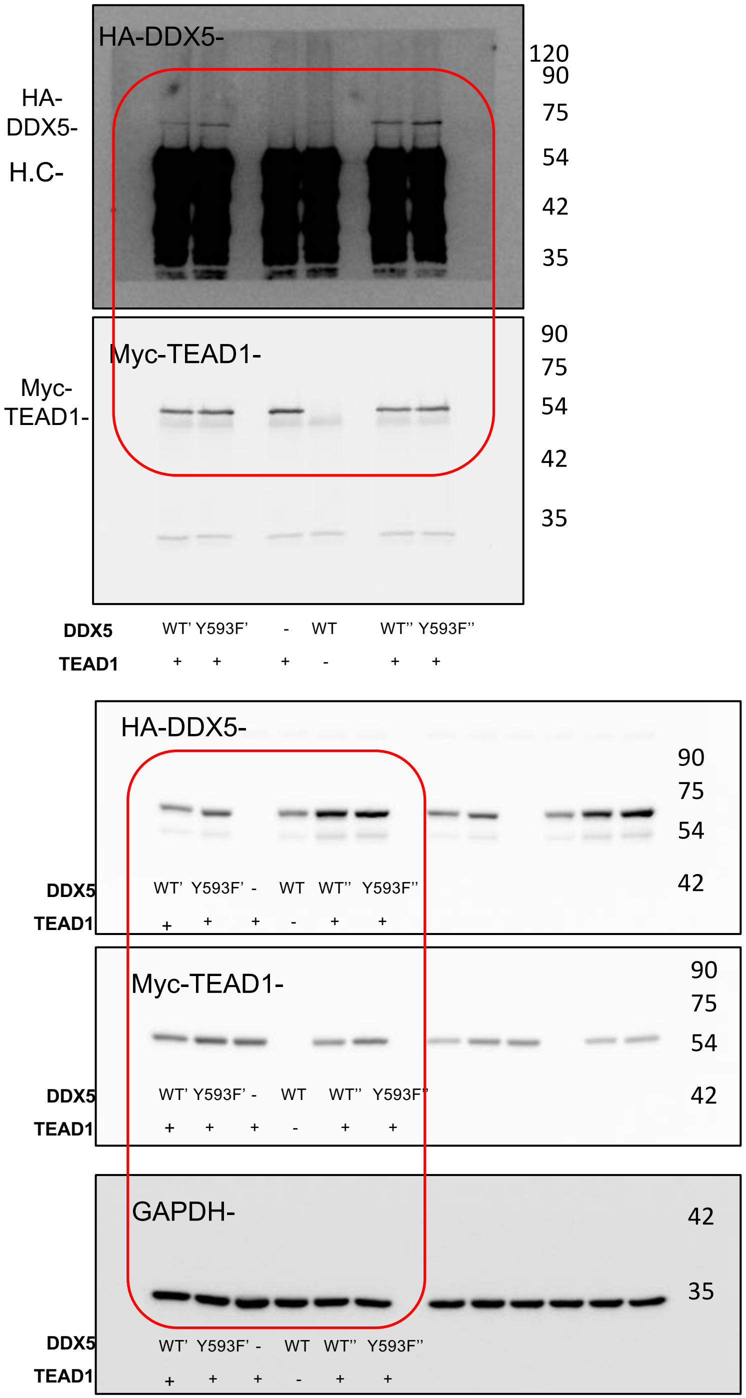

Figure S5H

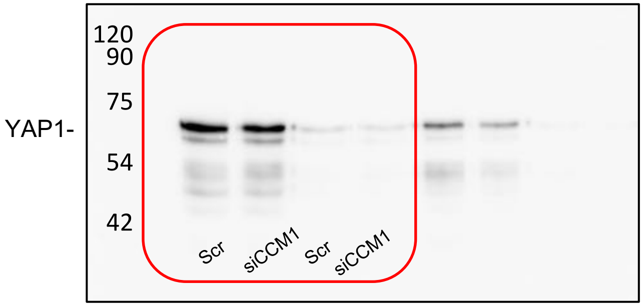

Figure S5I

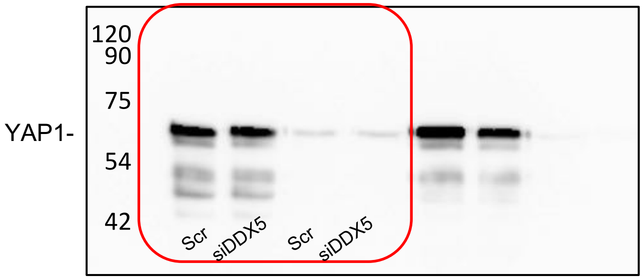

Figure S5K

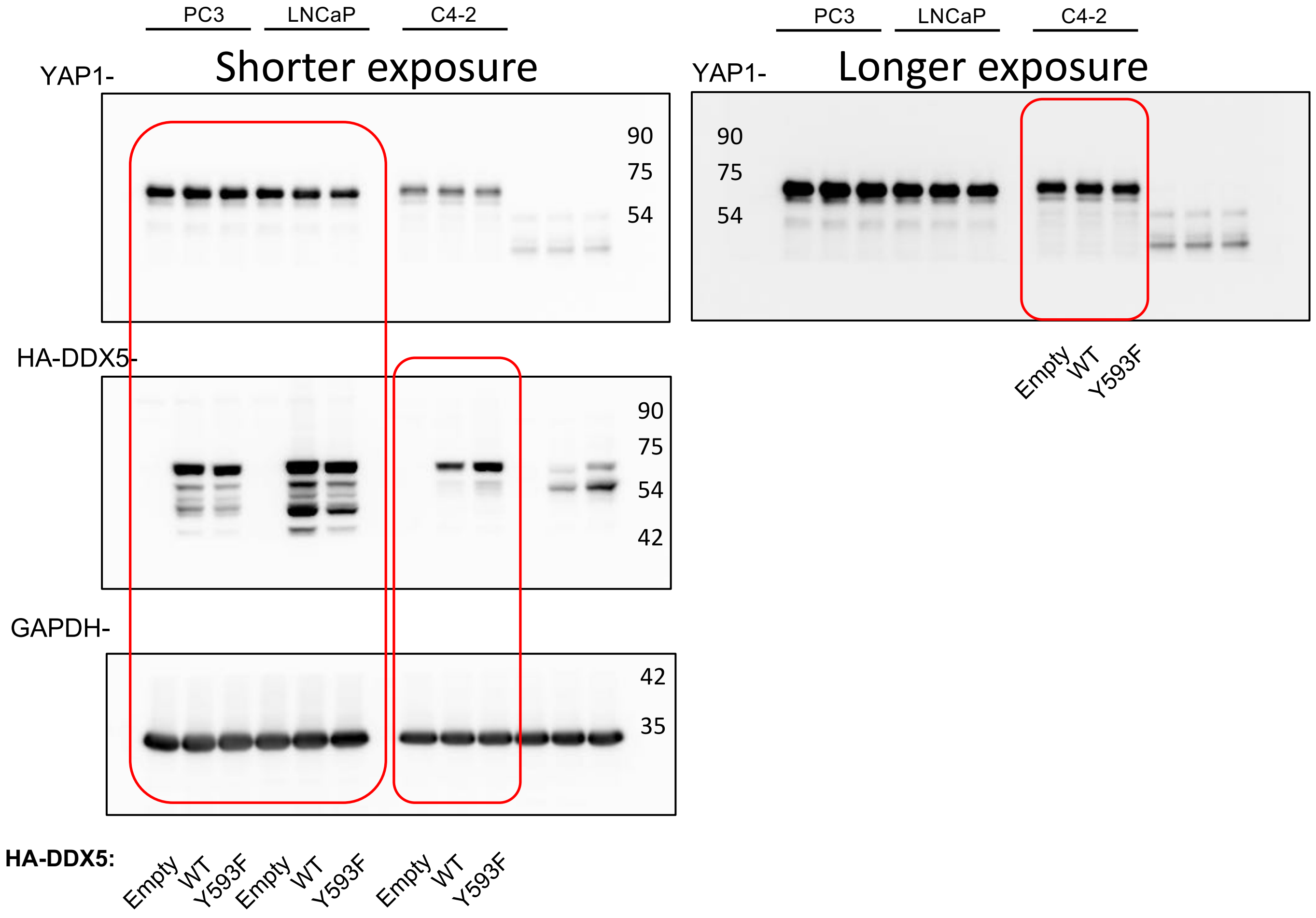

Figure S5L

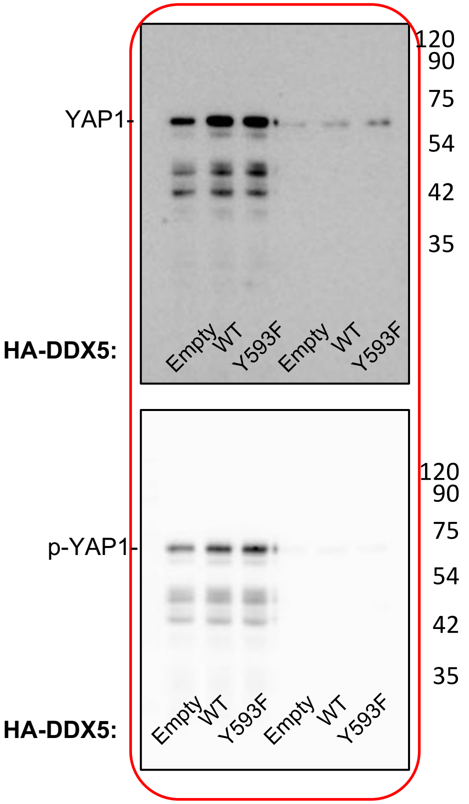

Supplement: Supplementary file 1 [file cancers-13-01125-s001.zip › cancers-1068035-supplementary file 2.pdf]
